# Optimal Application of Forced Air Warming to Prevent Peri-Operative Hypothermia during Abdominal Surgery: A Systematic Review and Meta-Analysis

**DOI:** 10.3390/ijerph18052517

**Published:** 2021-03-03

**Authors:** Yoonyoung Lee, Kisook Kim

**Affiliations:** 1 Department of Nursing, Sunchon National University, Jungang-ro, Suncheon, Jeonnam 57922, Korea; yylee@scnu.ac.kr; 2 Department of Nursing, Chung-Ang University, Heukseok-ro, Dongjak-gu, Seoul 06974, Korea

**Keywords:** peri-operative, hypothermia, meta-analysis, abdominal surgery, forced air warming, body temperature

## Abstract

Patients who undergo abdominal surgery under general anesthesia develop hypothermia in 80–90% of the cases within an hour after induction of anesthesia. Side effects include shivering, bleeding, and infection at the surgical site. However, the surgical team applies forced air warming to prevent peri-operative hypothermia, but these methods are insufficient. This study aimed to confirm the optimal application method of forced air warming (FAW) intervention for the prevention of peri-operative hypothermia during abdominal surgery. A systematic review and meta-analysis were conducted to provide a synthesized and critical appraisal of the studies included. We used PubMed, EMBASE, CINAHL, and Cochrane Library CENTRAL to systematically search for randomized controlled trials published through March 2020. Twelve studies were systematically reviewed for FAW intervention. FAW intervention effectively prevented peri-operative hypothermia among patients undergoing both open abdominal and laparoscopic surgery. Statistically significant effect size could not be confirmed in cases of only pre- or peri-operative application. The upper body was the primary application area, rather than the lower or full body. These findings could contribute detailed standards and criteria that can be effectively applied in the clinical field performing abdominal surgery.

## 1. Introduction

Peri-operative hypothermia refers to a state wherein the central body temperature is lowered to 36 degrees or less [1]. Peri-operative hypothermia occurs in more than 25–90% of patients [2] as a common postoperative complication with adverse effects, such as shivering, bleeding, coagulation disorder, surgical site infection, heart dysfunction, and delayed recovery during the postoperative recovery period [3]. Peri-operative hypothermia is induced by a wide variety of influencing factors, such as operation type, anesthesia type, anesthetic agent, operating room temperature, irrigation fluids, and intravenous fluids [4].

Particularly, in the case of abdominal surgery with general anesthesia without warming therapy, 27.6% of patients suffer from peri-operative hypothermia during the induction of anesthesia, 85.7% of patients from peri-operative hypothermia one hour after induction of anesthesia, and 88.6% of patients from peri-operative hypothermia at the end of anesthesia [5]. The cause of hypothermia during abdominal surgery is that after induction of general anesthesia, blood vessels dilate, followed by heat loss, leading to a decrease in body temperature [6]. In addition, it was reported that the body temperature further decreases as the abdominal cavity is exposed to the operating room environment [4].

To solve this problem, many experimental studies preventing peri-operative hypothermia have been started since the 1990s, and hypothermia has been minimized by active warming therapy [7]. Since study methods for systematic reviews and meta-analyses were introduced from the 2000s, many studies were conducted confirming the effectiveness of interventions to prevent peri-operative hypothermia. A systematic review was conducted confirming the effectiveness of peri-operative warming applied to patients undergoing surgery. The results were effective in preventing peri-operative hypothermia, shivering, surgical site infection, and postoperative pain [2,3,8]. In addition, as interventions to prevent peri-operative hypothermia in adult patients undergoing surgery, sheet, aluminum foil wrap, forced air warming, heat radiator, and intravenous fluid warming were used. Among these interventions, forced air warming was the most efficient method [9,10]. However, a recent study comparing the effects of four interventions to prevent peri-operative hypothermia noted no difference [11].

To confirm the effectiveness of active or passive warming techniques, most systematic reviews and meta-analyses used diverse subjects, types of surgery, and intervention methods in randomized controlled trials [9,12,13,14]. In a study, the subjects included patients who received neuraxial anesthesia [3]. A similar study was conducted on patients who received a cesarean section [15,16] and total hip and knee arthroplasty [17]. Systematic reviews confirming the effectiveness of interventions for the prevention of peri-operative hypothermia recommend warming intravenous and irrigation fluids [18] or using the circulating water garment system [19]. Various types of surgeries were also included in studies confirming the effectiveness of the forced-air warming system [20].

Most patients who underwent abdominal surgery under general anesthesia develop peri-operative hypothermia within an hour after anesthesia is started [5], but the method of applying forced air warming used to prevent peri-operative hypothermia is different. Therefore, presenting scientific evidence is crucial so that nurses can apply it effectively in clinical nursing practice.

Therefore, this study conducted systematic reviews and meta-analyses on the specific application method of forced air warming to prevent peri-operative hypothermia during abdominal surgery. The study provides detailed criteria of the procedure for effective application in clinical practice.

## 2. Materials and Methods

In this study, a systematic review and meta-analysis was performed according to the PRISMA (Preferential Reporting Items for Systematic Reviews and Meta-Analyses) guidelines [21] to confirm the optimal application methods of forced air warming for preventing peri-operative hypothermia during abdominal surgery.

### 2.1. Selection Criteria

#### 2.1.1. Inclusion Criteria

The key question considered for searching systematic literature review is, “Is forced air warming to prevent hypothermia in abdominal surgery patients more effective than general care?”. The study was conducted according to Populations, Intervention, Comparison, Outcome, and Study Design (PICOSD). Inclusion criteria included the following. The population (p) consisted of subjects who underwent abdominal surgery (stomach, duodenum, small intestine, colon, rectum, gallbladder, pancreas, peritoneum, kidney, uterus, abdominal aorta) under general anesthesia over 18 years of age; the intervention (I) was pre- or peri-operative forced air warming, the comparison (C) was with general care, the outcome (O) was the core temperature, and study design (SD) was all prospective randomized controlled trials (RCTs). We searched the literature of human subjects published up to 20 March 2020, and included all languages.

#### 2.1.2. Exclusion Criteria

Exclusion criteria included the following: (1) animal subject, neuraxial anesthesia, type of surgery (limb and spine surgery, thoracic surgery, outpatient surgery, obstetric surgery, plastic surgery, neurosurgery, and complex surgery), and surgery within 1 h; (2) irrigation fluid warming, intravenous fluid warming, multi intervention, postoperative warming, electric pad, towel wrapping, carbon fiber gown, full-body cover, heater, wrapping, operating room ambient temperature, etc.; and (3) conference abstracts without full-text articles, non-RCTs, systematic review and meta-analysis, guideline, reviews, letters, abstracts, editorials comments, or studies reporting insufficient data.

### 2.2. Search Strategy and Data Extraction Criteria

#### 2.2.1. Search Strategy

The search strategy for systematic review was developed and conducted by a literature search expert librarian experienced in systematic reviews with input from this study’s authors. On 20 March 2020, the search was conducted using the following electronic databases: PubMed, CINAHL (Cumulative Index for Nursing Allied Health Literature, EBSCO platform), Embase (Elsevier platform), and the Cochrane Central Register of Randomized Controlled Trials (Wiley platform). The search terms included hypothermia, warming, and body temperature. Search results were exported to EndNote^®^ X8 (Clarivate Analytics, Philadelphia, PA, USA), and duplicate articles were removed.

#### 2.2.2. Study Selection

Two researchers independently evaluated the search results, and after reviewing the title and abstract, the selected study underwent a full text review. The disagreement between the researchers was addressed through discussions, and if necessary, a third researcher evaluation.

#### 2.2.3. Data Extraction

The first researcher extracted data from the studies included in this research, and the second researcher confirmed the accuracy of the extraction. The disagreement between the two researchers was addressed through discussions. The data to be extracted from each selected study include the general characteristics of the study (first author, publication year, country, and study design), participants of the study (sample size and type of surgery), methods of intervention (devices, sites, and duration), and outcome of the study (site of the temperature, time points of measurements, and results about core temperature).

### 2.3. Quality Assessment

The version 2 (ROB 2) of Cochrane Collaboration’s risk-of-bias tool [22] was used to assess the quality of the selected randomized controlled trials. For each selected study, two researchers extracted and confirmed information on five domains: randomization process, deviations from the intended interventions, missing outcome data, measurement of the outcome, and selection of the reported result. Based on the RoB 2, the full text of each article was identified as exhibiting a “high risk”, “some concerns”, or “low risk.” Two reviewers independently evaluated the articles and discussed any differences to reach a consensus.

### 2.4. Statistical Analysis

In this study, a meta-analysis was performed when an outcome was reported in two or more studies and the study provided enough data to allow the calculation of effect sizes. If the study had multiple measuring points after intervention, effect sizes were primarily calculated with the end of surgery value. We estimated between-group standardized mean difference (SMD) with 95% confidence interval (CI) as the summary measure of effect and used means and standard deviations (SD) of outcomes to calculate the SMD. We used the I^2^ statistics to assess the heterogeneity of the included studies. If the I^2^ value was greater than 50%, the study was substantially heterogeneous, and a random effects model was applied to analyze the data [23].

However, we did not evaluate publication bias. According to the guidelines, tests for funnel plot asymmetry should be used only when a meta-analysis includes at least 10 studies, because with fewer studies, the power of the tests is too low to rule out chance in the observed asymmetry [23]. We conducted the meta-analysis with Cochrane Review Manager (RevMan, version 5.4.1; The Nordic Cochrane Centre, The Cochrane Collaboration, Copenhagen, Denmark) and considered *p*-values of less than 0.05 as statistically significant. All statistical tests were two-sided.

## 3. Results

### 3.1. Selected Studies

**Figure 1:**
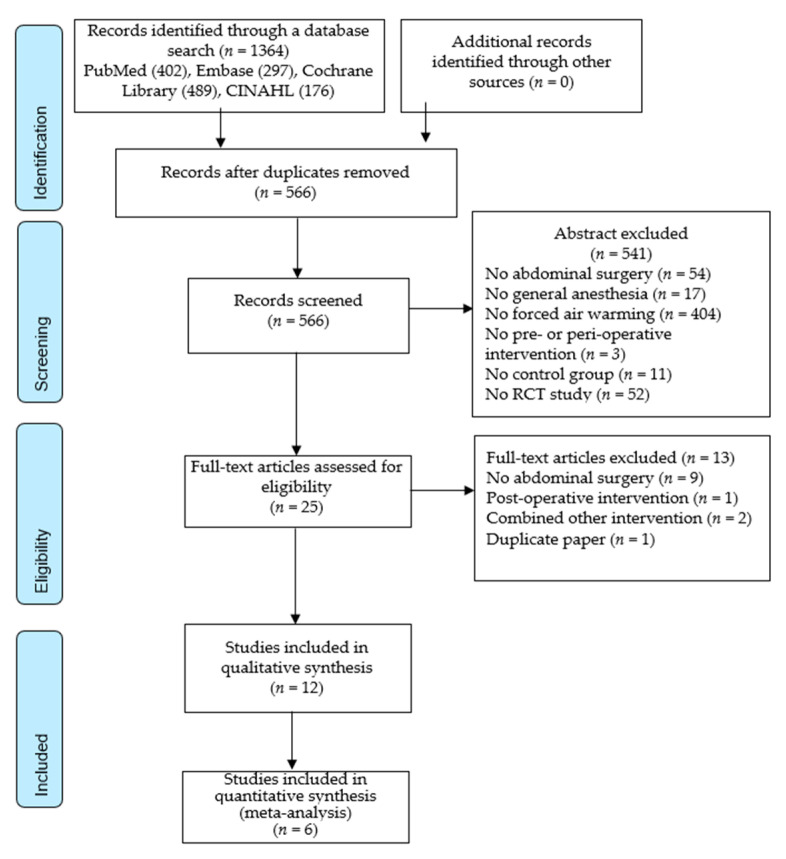
Preferred reporting items for systematic reviews and meta-analyses (PRISMA) flow.

The process of selecting studies was presented in Figure 1. Consequential to a search in four electronic databases, 1364 articles (402 in PubMed, 297 in EMbase, 268 in the Cochrane Library, and 176 in CINAHL) were examined. After the removal of 766 duplicate studies, 566 studies were screened to determine whether their titles and abstracts met the inclusion criteria. Consequentially, the full text of 25 studies were assessed for eligibility, and the final 12 studies were selected for systematic review and meta-analysis. The 13 excluded studies included nine non-abdominal surgery studies, one post-operative intervention, one combined other intervention in forced air warming, and one duplicate published study.

### 3.2. Study Characteristics

The characteristics of the 12 studies selected in the systematic review are summarized in Table 1. Eight studies were published before 2000 [24,25,26,27,28,29,30,31], and four were published after 2000 [32,33,34,35]. The first author’s country was Germany [24,30], Spain [25], France [26,27,29], Belgium [32], Sweden [34], USA [28,33], Japan [31], and China [35]. A total of 479 subjects participated in randomized controlled trials, and the number of participants per study ranged from 16 to 127.

### 3.3. Risk of Bias Assessment

**Figure 2:**
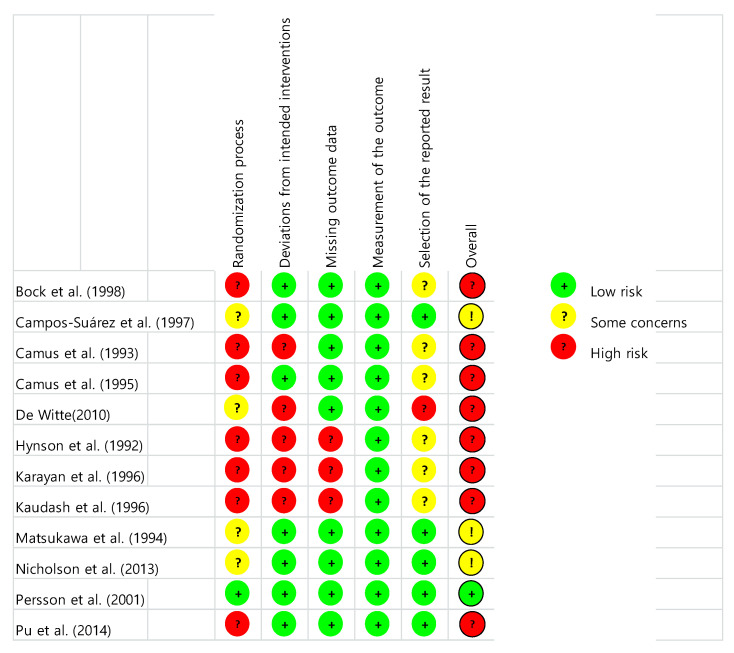
Risk of bias result.

**Figure 3:**
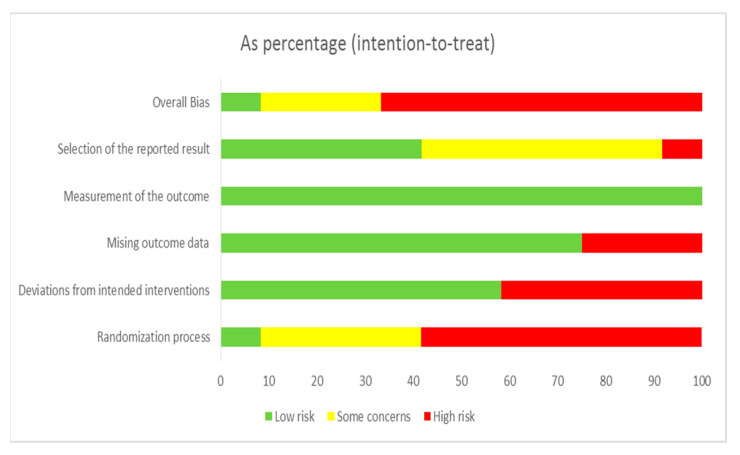
Risk of bias summary.

The 12 selected studies’ quality assessment results are presented in Figure 2 and Figure 3. “Overall bias” was evaluated as low risk of bias in only one study [34], and with some concerns or high risk in eleven studies. “Risk of bias arising from the randomization process” was evaluated as low risk of bias in only one study [34], and with some concerns or high risk in eleven studies. “Risk of bias due to deviations from intended interventions” was evaluated as low risk of bias in seven studies [24,25,27,31,33,34] and with high risk in five studies. “Risk of bias due to missing outcome data” was evaluated as low risk of bias in nine studies [24,25,26,27,31,32,33,34,35] and with high risk in three studies. “Risk of bias in measurement of the outcome” was evaluated as low risk of bias in all studies [24,25,26,27,28,29,30,31,32,33,34,35]. “Risk of bias in selection of the reported result” was evaluated as low risk of bias in five studies [25,31,33,34,35] and with some concerns or high risk in seven.

### 3.4. Intervention and Outcome Measures

The types of device used for forced air warming were Bear Hugger [25,26,27,28,29,31,35], warm touch system [24,30,34], and Arizant Healthcare Model 110 Peri-operative Blanket [32]. However, one study did not mention the type of device [33].

The application temperature of forced air warming was from 38 °C to 46 °C [24,26,27,28,30,31,32,34]. 

The application sites of forced air warming were the upper body [24,25,27,29,30,31,34], lower body [26,28,35], and full body [33].

The application durations of forced air warming were pre-operation [26,32,33], peri-operation [25,28,30,31,34,35], pre- and peri-operation [24,26], and activated when core temperature decreased to less than 36 °C [29].

### 3.5. Effects of Intervention and Subgroup Analysis

**Figure 4:**
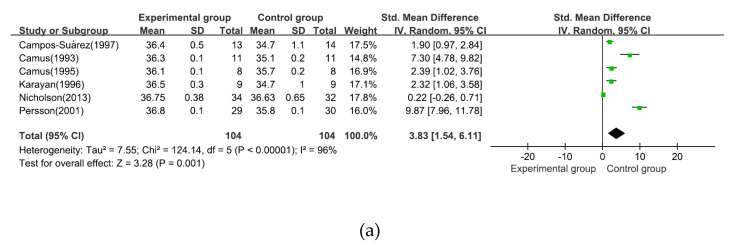

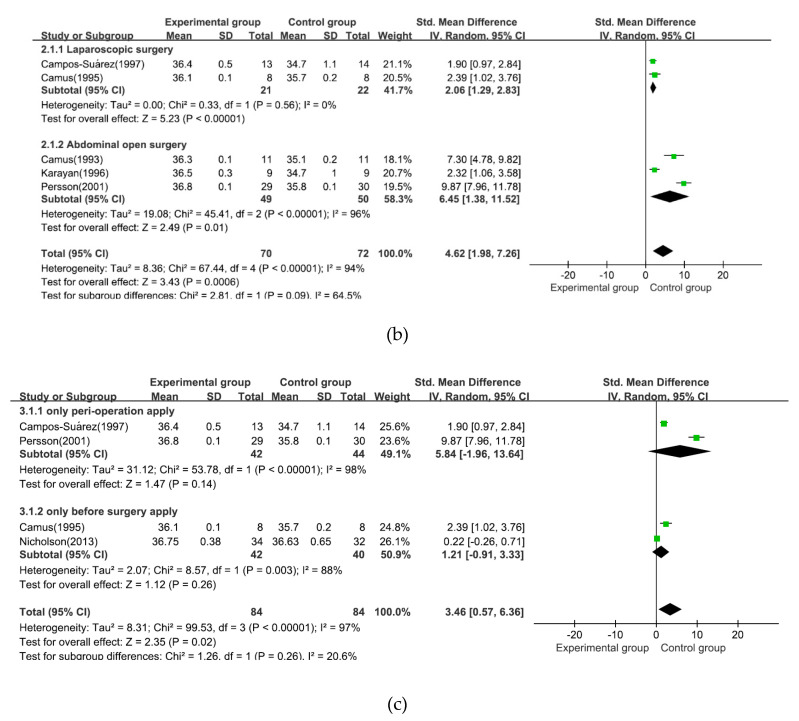
(**a**) Effect size of intervention; (**b**) subgroup analysis by surgery type; (**c**) application duration.

The effect sizes of the selected outcomes have been shown in Figure 4. Regarding measured outcomes, a statistically significant effect was noted on body temperature reduction prevention effect (z = 3.28, 95% CI [1.54, 6.11], *p* = 0.001, I^2^ = 96%). Subgroup analysis by surgery type (laparoscopic surgery vs. abdominal open surgery) showed statistically significant effects for both laparoscopic surgery (z = 5.23, 95% CI [1.29, 2.83], *p* = <0.001, I^2^ = 0%) and abdominal open surgery (z = 2.49, 95% CI [1.38, 11.52], *p* = 0.01, I^2^ = 96%). Another subgroup analysis by intervention application duration (only peri-operation application vs. only before surgery application) showed no statistically significant effects in both only peri-operation application (z = 1.47, 95% CI [−1.96, 13.64], *p* = 0.14, I^2^ = 98%) and only before surgery application (z = 1.12, 95%CI [−0.91, 3.33], *p* = 0.26, I^2^ = 88%).

## 4. Discussion

This study aimed to examine the existing literature about the specific application method of forced air warming to prevent peri-operative hypothermia during abdominal surgery. Further, it aimed to identify efficient evidence for assisting clinical practice by conducting systematic reviews and meta-analyses. 

Among the studies included in this study, 67% (eight studies) of studies were published prior to 2000. These results could indicate that many experimental studies on the effects of FAW have already been published, and the effects of the recent introduction of new interventions, such as Inditherm [36] and Orve + wrap [37]. However, comparative studies are being conducted to replace FAW. Further, as FAW is still commonly used in many cases, scientific suggestions are required regarding its usage in various types of surgery.

In this study, 479 participants were included in the FAW for prevention of peri-operative hypothermia during abdominal surgery. The number of study subjects was insufficient ranging from five to 55 per experimental group and control group. Even if the patient recruitment issues are considered in the patients involved in this study—and if enough subjects are included in the future study—it will play an important role in the calculation of the intervention effect size and an efficient presentation of evidence for the study synthesis results.

Consequential to the quality appraisal of the ROB of the selected study, overall bias was evaluated as low risk of bias in only one study, and it was a problem due to the lack of randomization and concealment. In future experimental research, an improved experimental research design will be required. The item of “Risk of bias in measurement of the outcome” was evaluated as low risk of bias in all studies. The method of measuring the core temperature as an outcome is not significantly affected by the bias of the subject and the measurer.

Among the studies included in this study, interventions using three types of FAW devices were employed in the experimental group, and the Bear Hugger was employed in seven studies. This can be a result of suggesting specific usage methods for commonly used instruments.

In eight studies presenting the warming temperature of the device during intervention, the temperature was maintained at 38–46 °C when FAW was applied. Most of them were found to be effective in preventing hypothermia during surgery. However, some studies did not suggest the warming temperature, and others only suggested the application temperature as “high”, “medium”, and “low” depending on the device. Therefore, to formulate clinical practice guidelines, it is crucial that the specific warming temperature before and during surgery and detailed guidelines for the warming temperature are provided.

This meta-analysis supports the efficacy of intervention in preventing hypothermia during abdominal surgery. Overall effect size of FAW intervention was statistically significant. In addition, these results are supported by several previous studies. Similar hypothermia prevention effects were verified in various types of surgery patients [9,13,20], particularly in total hip and knee arthroplasty [17]. However, the effects of FAW on outpatients showed different results than the current study and a previous study [38] that had no significant effect on hypothermia prevention. Since the operation time influences hypothermia during abdominal surgery [5], no significant effect was observed among outpatients with relatively short operation time; therefore, there is a difference. A total of five studies were included in the meta-analysis, and the overall bias was evaluated as high or there was a risk of bias in all five studies; this problem was caused by the lack of randomization and concealment. Except for one study, four studies were conducted in the 1990s. In most of these five studies, there was no mention of randomization or allocation concealment, or there was no method for randomization or concealment even if these were mentioned. Therefore, caution should be exercised when interpreting the results of this study, as there may be a risk of bias due to subjects being randomly assigned to a preferred intervention group.

Although only two and three studies were included in subgroup analysis for surgery type, both laparoscopic and open abdominal surgeries are effective in FAW. In addition, the effect size of abdominal open surgery is larger than laparoscopic surgery. Unlike laparoscopic surgery group, abdominal open surgery group’s heterogeneity is high. Prior studies have provided guidelines for the optimal time and method, and if hypothermia occurs during surgery [2,10,14] an appropriate intervention must be immediately provided, which proves to be challenge while recruiting many participants in a randomized experimental study.

Comparing the FAW intervention application time and duration, there were three pre-operative warming, six perioperative warming, and two pre- and peri-operative warming studies. In the previous study, the patient had to be actively prewarmed 20–30 min before surgery to counteract the decline in temperature [2]. Moreover, prewarmed patients must also be actively warmed intraoperatively, if the planned duration of anesthesia is longer than 60 min (without prewarming, 30 min). In another study, a 30-min warm-up was recommended along with the need to warm-up for more than 10 min before surgery [10], but it was different from the characteristics of the studies included in this research.

However, because of the subgroup analysis and meta-analysis performed in this study, “only peri-operation intervention application” and “only before surgery application” showed no significant effects. This might limit the results because in a few studies, insufficient data was available for statistical analysis. Several studies [28,30,31,35] researched the effects of only peri-operation FAW application, but the data was insufficient for the current study. In addition, in two studies [24,26], intervention was started before surgery and lasted until the end of the surgery. However, the effect size was not calculated because of insufficient availability of data. Since each individual study shows different results for the effectiveness of intervention, it is necessary to expand the scope of inclusion and study designs in the future, so that more accurate evidence can be discovered about the FAW intervention application time.

Regarding the application site, there were seven studies that applied FAW intervention to the upper body during abdominal surgery, three studies to the lower body, and two studies to the whole body. Thus, many studies applied the intervention to the upper body. In a study identifying the effect of FAW during hysterectomy, upper and lower body was compared in terms of warming and FAW application to prevent intra-operative hypothermia during hysterectomy under combined epidural and general anesthesia [39]. Additionally, in a study of randomized trial comparing FAW intervention to the upper and lower body for preventing hypothermia during thoracoscopic surgery, FAW application to prevent intraoperative hypothermia was found to be more effective on the lower body than on the upper body [40]. In this study, it was not possible to derive the effect size through meta-analysis due to the difference in measurement method and insufficient data. However, FAW should be applied to clinical practice in connection with previous research results.

In other studies, although FAW is an effective intervention in preventing hypothermia during surgery, no statistical evidence has been found proving its effectiveness compared with circulating-water garments, resistive heating blankets, and radiant warming systems [41]. However, FAW reduces the incidence of shivering and wound infections, increases thermal comfort, and reduces morbid cardiac event compared with other alternate form of warming and is commonly used in many institutions. [9,42]. Therefore, specific guidelines for patient-specific warming interventions during surgery should be developed as the basis for various types of surgery using FAW intervention.

Based on the results of this study, the following is suggested regarding the optimal application method of FAW for the prevention of peri-operative hypothermia during abdominal surgery. FAW is effective for the prevention of peri-operative hypothermia in laparoscopic surgery and laparotomy; therefore, it needs to be actively used in clinical practice. In addition, it is effective to actively begin applying FAW before the surgery and continue application during surgery rather than applying either before or during surgery. In future studies, the sample size should be larger and a randomized controlled trial protocol must be followed. In particular, we suggest conducting a study comparing the application of FAW in the upper body and in the lower body in abdominal surgery patients or a study to confirm the appropriate temperature.

### Limitations

Although this study contributes to the knowledge about FAW intervention for preventing hypothermia during abdominal surgery, it has several limitations. First, since a small number of studies (*n* = 6) were included in the meta-analysis stage, the interpretation of the results was limited. Second, the meta-analysis could not provide the effect size for some types of interventions due to insufficient data on interventions such as application temperature and site of FAW. If quantitative data could be presented through meta-analysis on the application site, application temperature, and application method for which meta-analysis was not performed due to insufficient data extraction for important variables, it would have contributed more to the development of detailed guidelines. In this study, the end of surgery was set as the outcome measurement point, but unfortunately, several studies suggested a decrease in body temperature as an outcome variable. Moreover, studies that were not included in the analysis included those wherein outcomes were measured at various time points, such as applying for one hour when the core body temperature decreases below a certain standard (36 degrees), or measuring until the time of admission to the recovery room or ICU. In addition, the heterogeneity of the studies included was high in both the overall effect and the subgroup analyses. Since the total number of studies and those in the subgroup analysis were small, the interpretation of the study results needs emphasis. Furthermore, heterogeneity increased due to the diverse subjects and the small number of participants. Finally, although we tried to retrieve all potentially eligible articles, we may have missed some.

## 5. Conclusions

Although several studies were conducted on SR and guidelines for the overall surgery regarding the effects of FAW intervention, few could provide specific evidence for the type of surgery with substantial diversity. In this study, the specific effect size of FAW intervention for the prevention of hypothermia in abdominal surgery patients was presented, and each intervention method, application time and timing, and application site were systematically reviewed and analyzed. FAW intervention was effective in preventing peri-operative hypothermia in abdominal surgery patients, and both open abdominal surgery and laparoscopic surgery were effective. In addition, many studies applied FAW intervention from the preoperative stage to the end of surgery, and statistically significant effect size could not be confirmed in only before surgery application and only peri operation application. For applying FAW, the upper body was used more than lower body and full body. Therefore, the findings of this study could contribute detailed standards and criteria for effective application in the clinical field of performing abdominal surgery. In addition, based on this study, a future study can be conducted to prepare specific guidelines for FAW intervention suited to various surgical methods and environment.

## Figures and Tables

**Table 1:** Descriptive summary of included studies.

	First Author, Publication Year, Country	Patients	Study Design	Intervention	Control Condition	Temperature Measurement	Outcome	Time Points of Measurements	Results about Core Temperature
1	Bock (1998) Germany	40 patients undergoing abdominal surgery Exp (*n* = 20) Cont (*n* = 20)	RCT	*Devices*: forced air warming (Warm touch system/42 °C) *Site*: upper body *Duration*: 30 min before induction of general anesthesia and during anesthesia	Passive protection against heat loss consisted of circulating water mattresses, blankets, and fluid warming device, which were used in both the Exp and Cont group	Tympanic membrane temperature	Primary outcome: core temperature differences Secondary outcome: shivering, blood loss, transfusion requirement, duration of stay in the PACU	After induction of anesthesia *Peri-operation*: 15-min intervals *Post-operation*: 30-min intervals	*15 min after intubation to ~ end of surgery* Exp 0.5 (0.8) Cont 1.5 (0.8) *p* < 0.01 *180 min after arrival PACU* *p* < 0.01
2	Campos-Suárez (1997) Spain	30 patients undergoing laparoscopic abdominal surgery Exp (*n* = 13) Cont (*n* = 14)	RCT	*Devices*: forced air warming (Bear Hugger) *Site*: upper body (chest and arms) *Duration*: started after induction of anesthesia until end of surgery	Normal care	Esophageal temperature	Primary outcome core temperature differences	*Peri-operation*: 30-min intervals	*End of surgery* Exp 36.4 (0.5) Cont 34.7 (1.1) *p* < 0.0001 *After arrival PACU* Exp 36.3 (0.65) Cont 34.87 (1.0)
3	Camus (1993) France	33 patients undergoing abdominal surgery Exp1 (*n* = 11) Cont1 (*n* = 11) Cont2 (*n* = 11)	RCT	*Devices*: forced air warming (Bear Hugger/43 °C) *Site*: lower body *Duration*: After lying on the operating table and during anesthesia	Cont1: cotton blanket Cont2: forced air warming+ cotton blanket	Core temperature	Primary outcome: core temperature differences Secondary outcome: shivering occurrence	After lying on the operating table *Peri- and post-operation*: 30-min intervals	*End of surgery* Exp 0.4 (0.1) Cont 1.8 (0.2) *p* < 0.001
4	Camus (1995) France	16 patients scheduled for laparoscopic cholecystectomy Exp (*n* = 8) Cont (*n* = 8)	RCT	*Devices*: forced air warming (Bear Hugger/41 °C) *Site*: upper body (covered up to the shoulder) *Duration*: one hour before induction of anesthesia (pre-induction period)	Wool blanket	Tympanic membrane temperature	Primary outcome: pre-warmed groups efficacy, shivering	From patient’s arrival in the pre operating area *Pre and peri-operation*: 15-min intervals	*After induction of anesthesia* Exp 0.6 (0.1) Cont 1.1 (0.1) *p* < 0.05 *After one hour of anesthesia* Exp 36.6 (0.1) Cont 36.0 (0.1) *p* < 0.05 *End of surgery* Exp 36.1 (0.1) Cont 35.7 (0.2) *p* < 0.05
5	De Witte (2010) Belgium	26 patients scheduled for laparoscopic colorectal surgery Exp (*n* = 9) Cont1 (*n* = 8) Cont2 (*n* = 9)	RCT	*Devices*: forced air warming (Arizant Healthcare Model 110 Peri-oprative Blanket /42 °C) *Site*: cover excluded the shoulders, ankles, and feet *Duration*: during 30 min before induction of anesthesia	Cont1: no prewarming Cont2: carbon fiber total body cover	Esophageal temperatures Tympanic membrane temperature	Primary outcome: intraoperative core temperature	10 min before prewarming and continued until discharge from the post anesthesia care unit (PACU) *Pre-, Peri-, and post-operation:* 5-min intervals	*End of the prewarming period* Exp 35.9 (0.5) Cont1 35.9 (0.5) *After 50 min of anesthesia* Exp 36.2 (0.35) Cont1 35.9 (0.3) *60 min after arrival at PACU* Exp 35.5 (0.8) Cont1 35.4 (1.0)
6	Hynson (1992) USA	20 patients undergoing kidney transplantation for end-stage renal disease Exp (*n* = 5) Cont1 (*n* = 5) Cont2 (*n* = 5) Cont3 (*n* = 5)	RCT	*Devices*: forced air warming (Bear Hugger/43 °C) *Site*: lower body *Duration*: during anesthesia	Cont1: cotton blanket Cont2: circulating-water blanket (40 °C), Cont3: heated humidifier (40 °C),	Tympanic membrane temperature	Primary outcome: core temperature differences Secondary outcome: cutaneous heat loss	After induction of anesthesia (during 3-h) *Peri- and post-operation*: 10-min intervals	*After 1 h of anesthesia* decreased 1 °C in all groups *After 2 h of anesthesia* Only Exp remains constant *After 3 h of anesthesia,* Exp (−0.5 °C ± 0.4 °C), Cont1 (−2.0 °C ± 0.7 °C) Cont2 (−1.2 °C ± 0.4 °C) Cont3 (−2.0 °C ± 0.5 °C)
7	Karayan (1996) France	18 patients undergoing abdominal aortic surgery Exp (*n* = 9) Cont (*n* = 9)	RCT	*Devices*: forced air warming (Bear Hugger) *Site*: upper body (upper chest and arms) *Duration*: activated when core temperature decreased to less than 36 °C	Warm cotton sheet	Pulmonary artery catheter temperature	Primary outcome: efficacy of delayed warming system	*Peri-operation*: 1-h intervals	*After 1 h of anesthesia* Both groups decreased (0.6 °C) *After 2 h of anesthesia* Both groups decreased (0.4 °C) *End of surgery* Exp 36.5 (0.32) Cont 34.7 (1.0) *p* < 0.003
8	Kaudasch (1996) Germany	24 patients scheduled for colon surgery Exp (*n* = 12) Cont (*n* = 12)	RCT	*Devices*: forced air warming (Warm touch system/46 °C) *Site*: upper body *Duration*: started after induction of anesthesia	Cotton blanket	Esophageal and urinary bladder temperature	Primary outcome: core temperature differences Secondary outcome: heat loss of skin, shivering	*Peri-operation*: arrival in the OR, skin incision, peritoneal incision, anastomosis, and skin suture *Post-operation*: administration to the ICU (each 30 min for 180 min)	*End of surgery* Exp 36.3 °C Cont 35.2 °C *Administration to the ICU* Exp 36.2 °C Cont 35.4 °C No statistical difference
9	Matsukawa (1994) Japan	40 patients with open abdominal surgery Exp (*n* = 20) Cont (*n* = 20)	RCT	*Devices*: forced air warming (Bear Hugger/38 °C) *Site*: upper body (thoracic region and upper limbs) *Duration*: during anesthesia	Circulating blanket warming was used both in the Exp and Cont group	Rectal temperatures	Primary outcome: core and digital temperature differences Secondary outcome: shivering occurrence	After the start of operation *Peri-operation*: 30-min intervals (0, 30, 60, 90, 120, 150, 150-min)	*Peri-operation* *p* < 0.01
10	Nicholson (2013) USA	66 patients undergoing colorectal surgery (laparoscopic and open surgery) Exp (*n* = 34) Cont (*n* = 32)	RCT	*Devices*: forced air warming *Site*: whole body (gown covered upper and lower body) *Duration*: at least 30 min in the preoperative setting	Cotton blanket	Oral temperature (pre- and post- operative) Esophageal, rectal and urinary bladder temperature (peri operative)	Primary outcome: efficacy of prewarming	*Pre-operation*: 30-min or longer after warming *Peri-operation*: after intubation *Post-operation*: 15-min after surgery in the PACU	*Pre-operation* Exp 36.80 (0.21) Cont 36.76 (0.22) *p* = 0.419 *Peri-operation* Exp 36.12 (0.65) Cont 35.88 (0.60) *p* = 0.126 *Post-operation * Exp 36.75 (0.38) Cont 36.63 (0.65) *p* = 0.353
11	Persson (2001) Sweden	59 patients undergoing subtotal hysterectomy Exp (*n* = 29) Cont (*n* = 30)	RCT	*Devices*: forced air warming (Warm touch system/43~46 °C) *Site*: upper body (chest adjacent to the skin covering both arms) *Duration*: started after induction of anesthesia and stopped at the end of operation.	Cotton blankets	Tympanic membrane	Primary outcome: core temperature differences Secondary outcome: analgesic requirement, pain intensity, blood loss	*Peri-operation*: 15-min intervals (0~120 min) *Post-operation*: 15-min intervals (0~210)	*105 min after induction* Exp 36.8 (0.1) Cont 35.8 (0.1) *p* = 0.001 *End of surgery* Exp: regained the preinduction temperature Cont: not reach 36.5 °C until 180 min
12	Pu (2014) China	110 patients undergoing laparoscopic gastrointestinal surgery. Exp (*n* = 55) Cont (*n* = 55)	RCT	*Devices*: forced air warming (Bear Hugger) *Site*: underbody *Duration*: during anesthesia	Quilt	Nasopharyngeal temperature	Primary outcome: hypothermia during operation Secondary outcome: shivering after anesthesia, complication, such as hemorrhage, coagulation functions, and pain level	*Pre-operation*: before anesthesia *Peri-operation*: right after anesthesia, right after start of the operation *Post-operation*: 10 min thereafter until the end of anesthesia	*Peri-operation* Exp 3/55 (5.5%) Cont 29/55 (62.7%) *p* < 0.001 No significant alteration in the temperature at the beginning of surgery until 30 min later decreased beginning from 30 min after the start of surgery until the end of surgery *p* < 0.001–0.05

RCT, randomized controlled trial; Exp, experimental group; Cont, control group.

## Data Availability

The data that support the findings of this study are available from the corresponding author, upon reasonable request.
